# Combined basilar subsegmentectomy for intralobar sequestration via uniportal VATS: a case report

**DOI:** 10.1186/s40792-023-01600-3

**Published:** 2023-02-01

**Authors:** Satoshi Takamori, Hiroyuki Oizumi, Jun Suzuki, Satoshi Shiono

**Affiliations:** 1 Department of Surgery II, Faculty of Medicine, Yamagata University, 2-2-2 Iida-Nishi, Yamagata, 990-9585 Japan; 2 Department of General Thoracic Surgery, Higashiyamato Hospital, 1-13-12 Nangai, Higashiyamato, Tokyo 207-0014 Japan

**Keywords:** Intralobar pulmonary sequestration, Subsegmentectomy, Uniportal video-assisted thoracoscopic surgery

## Abstract

**Background:**

Combined basilar subsegmentectomy via uniportal video-assisted thoracoscopic surgery is an extremely complex surgery. Moreover, no the existing reports describe the procedure and technique. Here, we present the technique of combined basilar subsegmentectomy that was successfully performed via uniportal video-assisted thoracoscopic surgery to treat intralobar pulmonary sequestration in an adult patient.

**Case presentation:**

A 57-year-old man underwent surgery for oropharyngeal carcinoma. Preoperative computed tomography showed several cystic lesions in the right lower lobe. Subsequent enhanced computed tomography revealed an anomalous artery branching from the abdominal aorta and a normal pulmonary vein. The patient with diagnosed with Pryce type III intralobar pulmonary sequestration and underwent right S7 posterior + 10bc combined basilar segmentectomy via uniportal video-assisted thoracoscopic surgery. The postoperative course was uneventful, and the patient was discharged 4 days after surgery. At the 8-month follow-up, computed tomography showed no abnormalities.

**Conclusions:**

We successfully performed combined basilar subsegmentectomy via uniportal video-assisted thoracoscopic surgery. This surgical approach is useful for the treatment of intralobar pulmonary sequestration occurring at the basal segment of the lung.

**Supplementary Information:**

The online version contains supplementary material available at 10.1186/s40792-023-01600-3.

## Background

Intralobar pulmonary sequestration (ILPS) is characterized by a nonfunctional lung segment within the normal pulmonary parenchyma, which is supplied by an anomalous systemic artery [1]. The definitive treatment for ILPS is surgical excision; however, for benign disease, anatomical segmentectomy using a minimally invasive approach may be sufficient if the entire sequestered tissue is included in the segment [2]. Combined basilar subsegmentectomy via uniportal video-assisted thoracoscopic surgery (U-VATS) is an extremely complex surgery; moreover, to the best of our knowledge, there are no reports describing the procedure. Herein, we present the technique of combined basilar subsegmentectomy successfully performed via U-VATS to treat ILPS in an adult patient.

## Case presentation

**Fig. 1:**
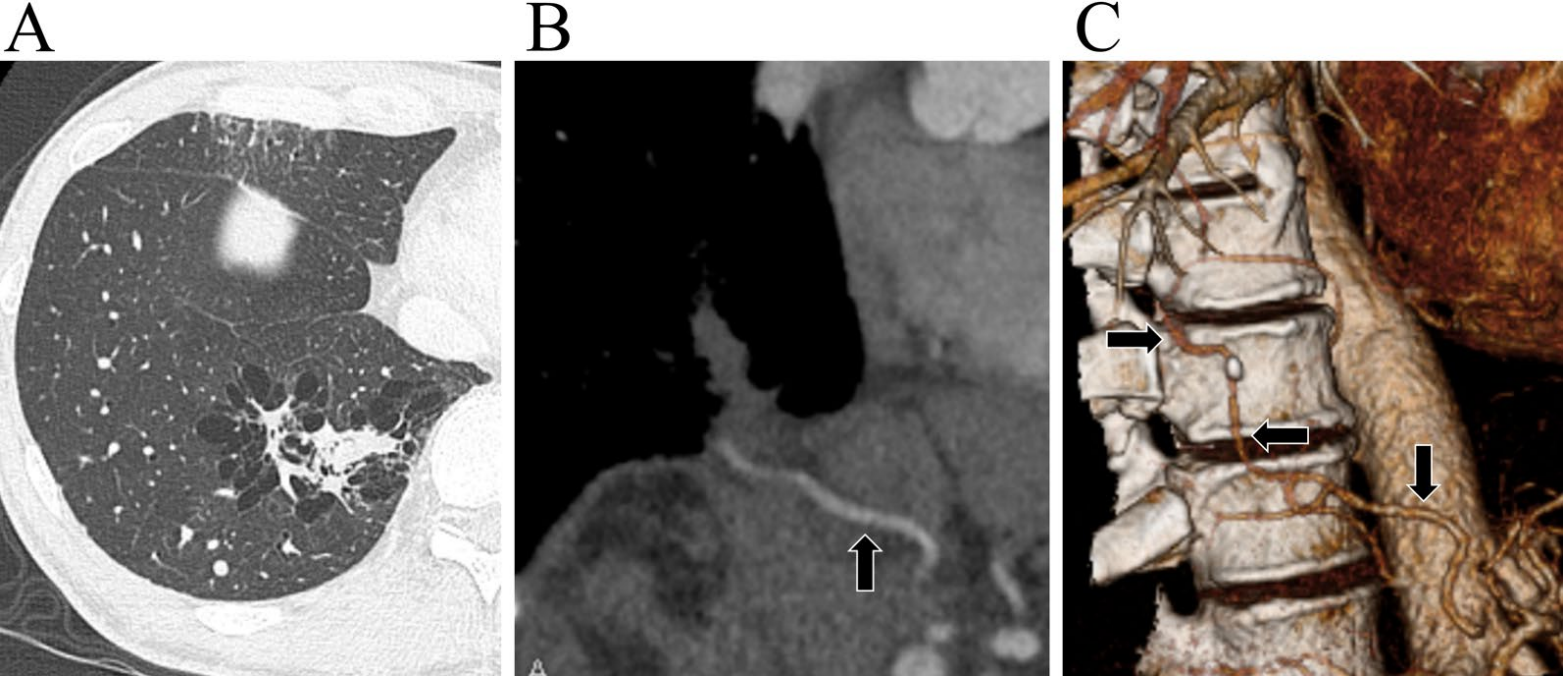
Preoperative image of enhanced computed tomography and three-dimensional computed tomography. **A** Enhanced computed tomography (CT) scan showing intralobar sequestration. **B** Enhanced CT scan reveals an abnormal artery branching from the abdominal aorta (arrow). **C** Three-dimensional CT scan reveals an abnormal artery branching from the abdominal aorta (arrow)

**Fig. 2:**
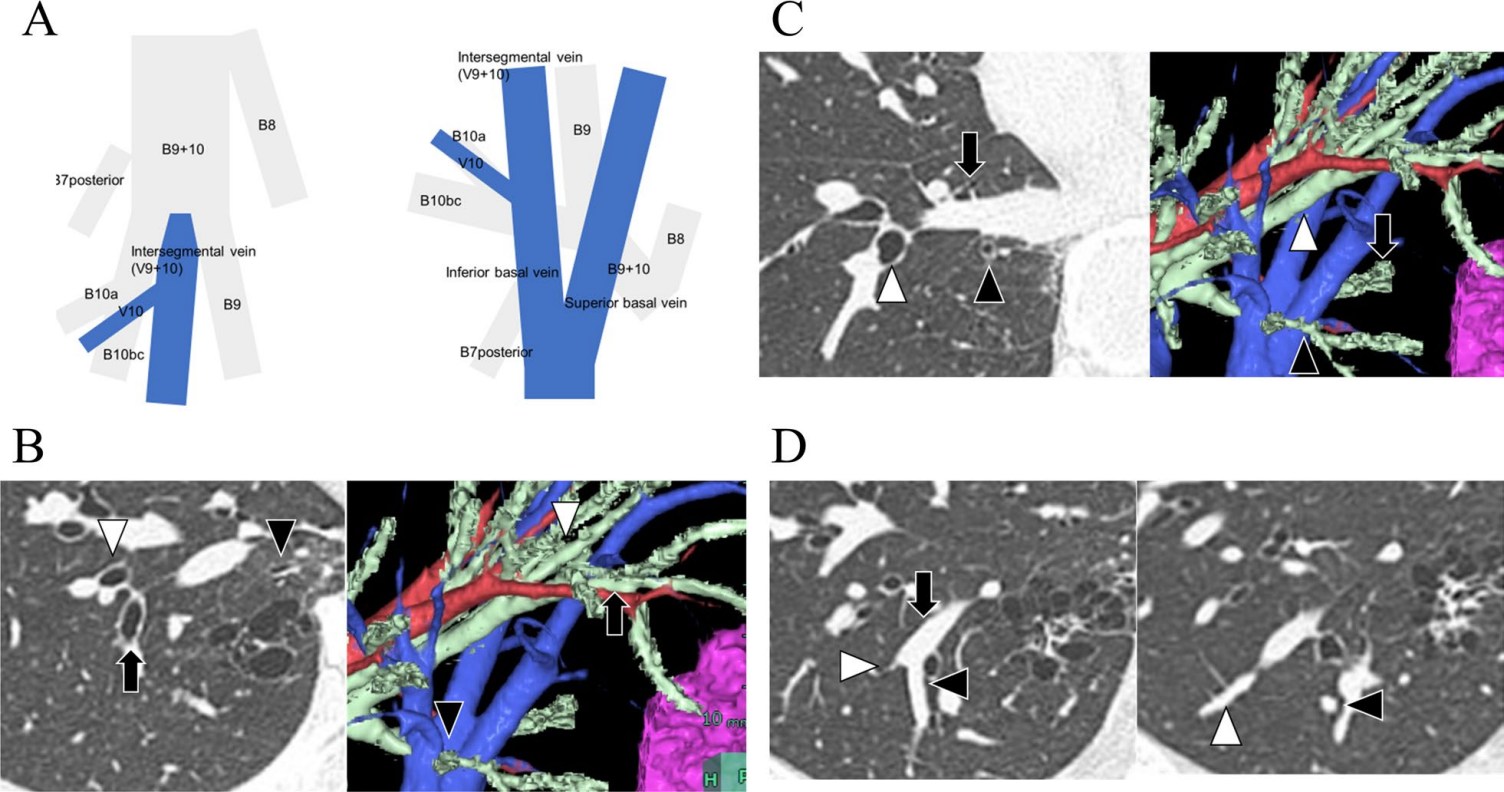
Schema of the anatomy of the basilar segment and intraoperative image. **A** Schematic of conventional and surgeon’s views of the vein and bronchus. **B** B7 posterior (black arrowhead), B7 anterior (arrow), and B9 + 10 (white arrowhead) are shown. **C** The B7 posterior (black arrowhead), B10bc (arrow), and B9 (white arrowhead) are shown. For complete resection of the lesion, it was necessary to divide the B10bc from the B7 posterior. **D** The V9 + 10 (arrow) is shown. Intersegmental vein preservation between S9 and S10 (white arrowhead) and division of the intrasegmental vein (black arrowhead) were planned

A 57-year-old man underwent surgery for oropharyngeal carcinoma. Preoperative computed tomography (CT) revealed several cystic lesions in the right lower lung lobe (Fig. 1A) with consolidation and without direct communication with the bronchus. Enhanced and three-dimensional reconstruction CT (3D-CT) revealed an anomalous artery branching from the abdominal aorta and a normal pulmonary vein (Fig. 1B, C); thus, our diagnosis was Pryce type III ILPS. Laboratory findings were normal without any evidence of inflammation. The B7 bronchus branched anteriorly and posteriorly above the common basilar vein. The lesion was limited within the S7 posterior and S10bc, with normal remnant parenchyma. We planned a right S7 posterior + 10bc combined basilar subsegmentectomy via U-VATS based on the CT and 3DCT findings (Fig. 2A–D).

**Fig. 3:**
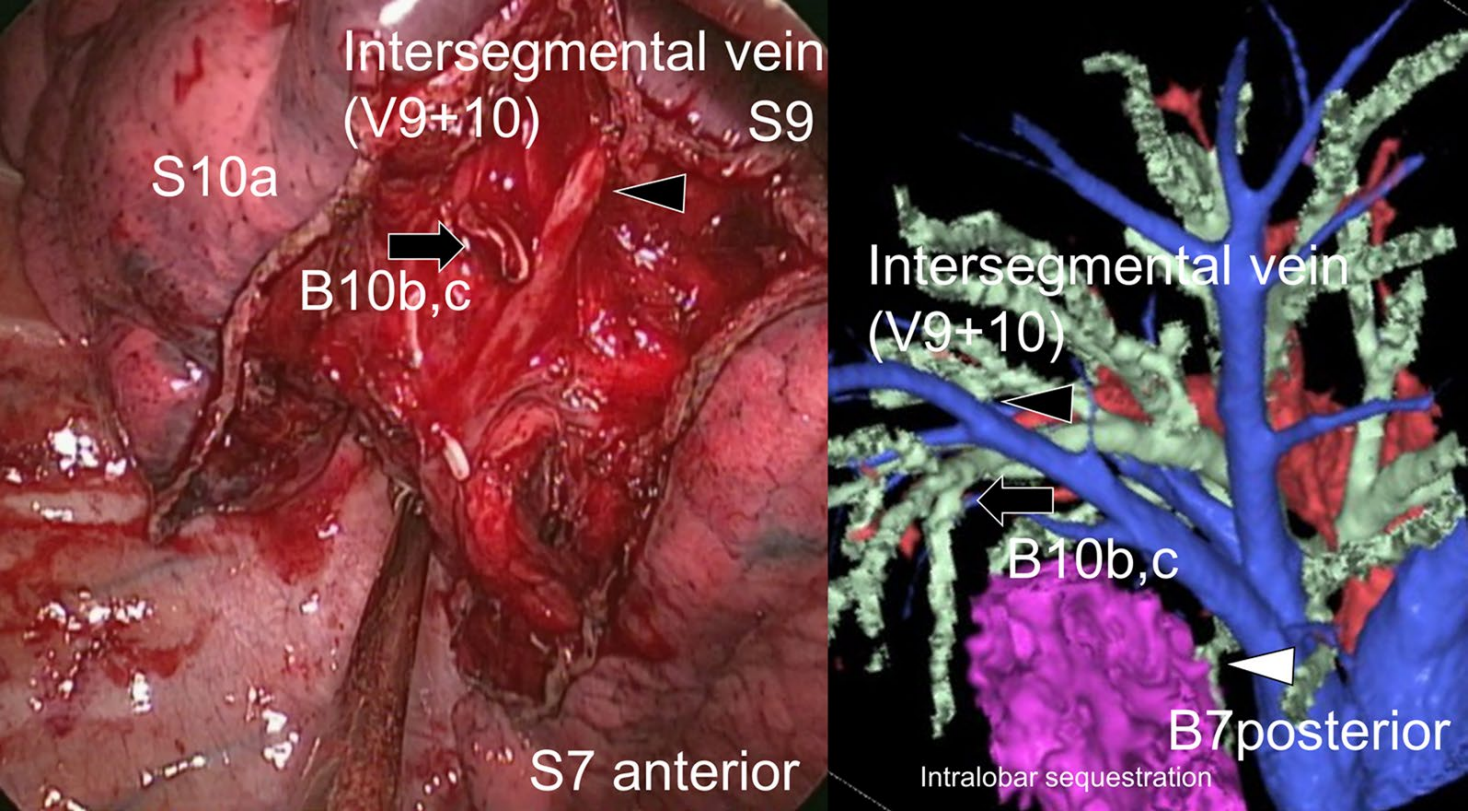
Thoracoscopic combined basilar subsegmentectomy (S7 posteror + 10bc) for intralobar sequestration

**Fig. 4:**
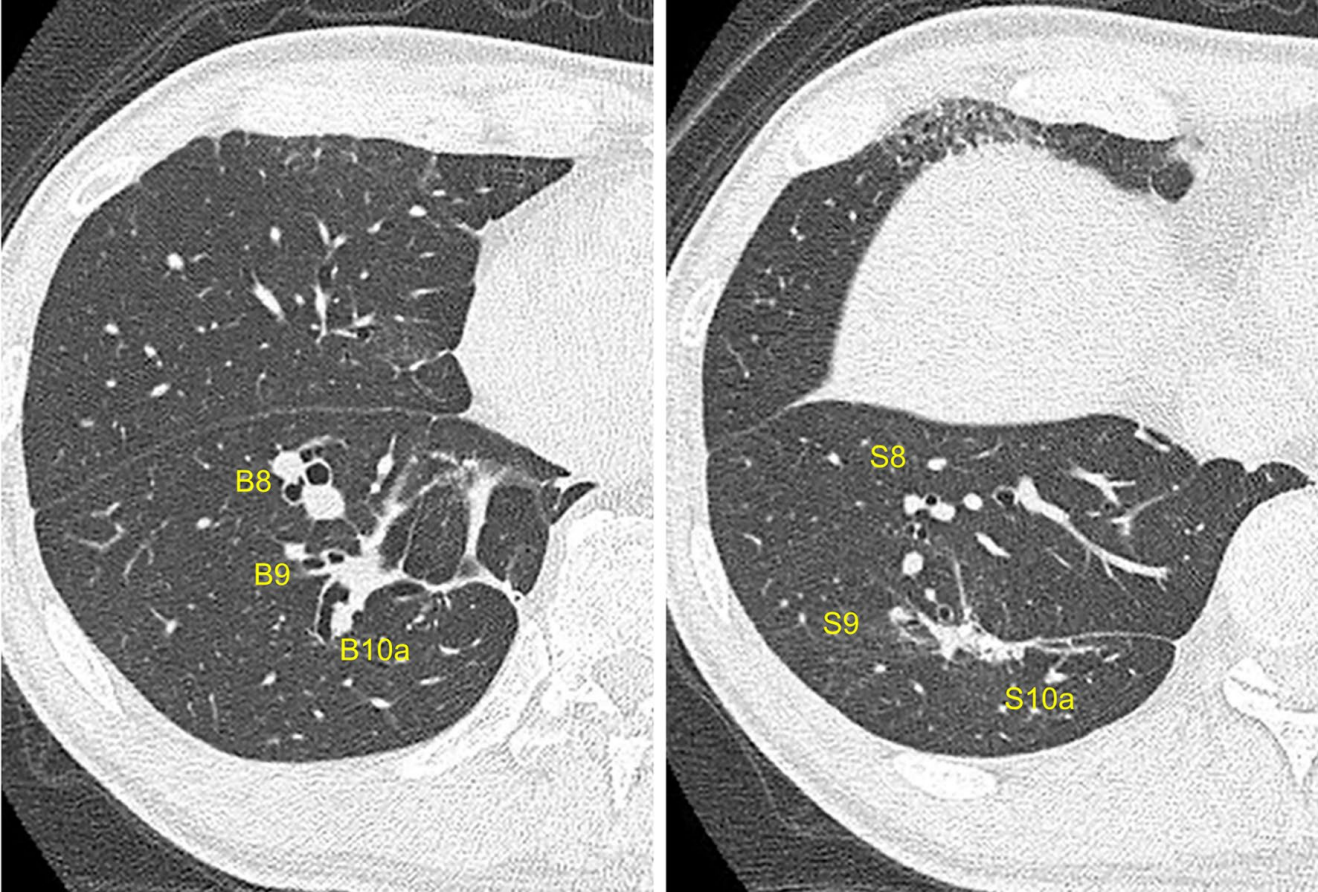
Postoperative image of computed tomography

We made 4-cm utility incision along the midaxillary line in the sixth intercostal space. After adhesiolysis, an anomalous artery from the abdominal aorta in the lung ligament was ligated and divided. We dissected the lung parenchyma to expose V7. Subsequently, the plane between S7 anterior and S7 posterior was opened using a stapler along the V7 (intersegmental vein between S7 anterior and S7 posterior). The B7 posterior was identified beside the inferior basal pulmonary vein and was clipped and divided. Subsequently, the A7 posterior was identified and divided using a vessel-sealing system without proximal ligation. We meticulously dissected the pulmonary parenchyma along the inferior basal pulmonary vein, with the B10bc identified distally and divided using a stapler. After dividing these hilum structures, the pulmonary parenchyma was divided using the stapler. Thereafter, S7 posterior + 10bc combined basilar subsegmentectomy was performed (Fig. 3; Additional file 1: Video S1). The operation time was 202 min, and the total blood loss was 180 mL. The postoperative course was uneventful, and the patient was discharged 4 days after surgery. Histopathologic examination confirmed the diagnosis of ILPS. At the 8-month postoperative follow-up, CT revealed no abnormalities (Fig. 4). The case report was approved by the Ethics Committee of the Faculty of Medicine, Yamagata University (#2020-S-60, December 15, 2020). Informed consent to publish this report was obtained from the patient.

## Discussion

The primary treatment for ILPS is surgical resection. The feasibility of segmentectomy via VATS for ILPS has been demonstrated previously [2, 3]. We consider segmentectomy to be an adequate treatment for benign disease if the lesion is included in the segment and can be removed completely. A potential advantage of segmentectomy over lobectomy is its improved capacity to preserve pulmonary function. Here, we performed an anatomically combined subsegmentectomy via U-VATS; therefore, the preserved lung maintained considerable volume, likely facilitating the preservation of a substantial degree of pulmonary function.

In this case, combined basilar subsegmentectomy via U-VATS was successfully used to treat ILPS in an adult patient. Nevertheless, U-VATS is considered technically difficult due to interference among instruments and the limited insertion angles caused by inserting all instruments including the thoracoscope through a single small wound. However, a previous study has reported U-VATS for treating ILPS and that uniportal VATS is comparable to multiportal VATS in terms of blood loss, surgical time, and postoperative outcomes [2]. Interestingly, a recent report revealed that U-VATS segmentectomies are better than multiportal VATS segmentectomies in postoperative outcomes [4, 5]. Factors including postoperative symptom relief and recovery time for U-VATS are encouraging.

In this case, preoperative 3D-CT was performed to determine the precise anatomy of complex vessels and bronchi. The precise interpretation of the structure of the target lung segment and its review from various angles before the surgery using enhanced CT and 3D-CT are essential. In parenchyma-sparing resection for ILPS, where resection of complex segmental structures or peripheral dissection of the pulmonary vein is required, preoperative 3D-CT is helpful for strategic surgery planning [6]. Although it is difficult to move the lung with U-VATS basilar subsegmentectomy, the technique may be used to dissect deep segment structures such as those described in this case. In limited resection involving the S7 posterior and S10bc, hilar structures are very deep from the interlobar fissure and require disrupted dissection of the lung parenchyma. However, the S7 posterior and S10bc bronchi are shallow for the non-fissure approach, making them easily accessible via a vein-first surgical strategy [7]. Furthermore, careful peripheral dissection of the vascular sheath of the pulmonary vein facilitates easy identification of the bronchi. The segmental plane was divided after pulmonary hilar structures were processed. Previous studies [7–12] have indicated that this approach might be useful not only for patients with a benign disease but also for those with metastatic lung tumors or small-sized non-solid lung cancer and those who underwent only passive limited resection for lung cancer. However, when lymph node dissection is performed, using the vein-first strategy might make it difficult to adequately approach the pulmonary hilum. Therefore, this method might be carefully indicated when lymph node dissection is required.

## Conclusions

Although the indications for combined subsegmentectomy via U-VATS might be limited, factors including postoperative symptom relief and recovery time for U-VATS are encouraging, and it is important for patients that large lungs remain. This method should be considered as an important surgical approach for treating ILPS.

## Supplementary Information

**Additional file 1: Video S1.** Combined basilar subsegmentectomy (S7 posterior + S10bc) for intralobar sequestration via uniportal video-assisted thoracoscopic surgery.

## Data Availability

Not applicable.

## Abbreviations

CT: Computed tomography
3D-CT: Three-dimensional reconstruction computed tomography
ILPS: Intralobar pulmonary sequestration
U-VATS: Uniportal video-assisted thoracoscopic surgery
